# Etanercept restores normal insulin signal transduction in β2-adrenergic receptor knockout mice

**DOI:** 10.1186/s12974-014-0137-z

**Published:** 2014-08-20

**Authors:** Youde Jiang, Qiuhua Zhang, Eun-Ah Ye, Jena J Steinle

**Affiliations:** Department of Ophthalmology, University of Tennessee Health Science Center, Memphis, TN USA; Department of Anatomy & Neurobiology, University of Tennessee Health Science Center, Memphis, TN USA; Department of Pharmaceutical Sciences, University of Tennessee Health Science Center, Memphis, TN USA; Department of Ophthalmology, Hamilton Eye Institute, 930 Madison Ave, Suite 768A, Memphis, TN 38163 USA

**Keywords:** TNFα, SOCS3, Apoptosis, Insulin signaling

## Abstract

**Background:**

Inhibition of TNFα protects the retina against diabetic-like changes in rodent models. The mechanism by which TNFα induces deleterious retinal changes is not known. Previously, we have shown that TNFα can inhibit normal insulin signal transduction, leading to increased apoptosis in both retinal endothelial cells (REC) and Müller cells. Additionally, β2-adrenergic receptor knockout mice (β2KO) have increased TNFα levels and decreased insulin receptor activity. In this study, we hypothesized that inhibition of TNFα in β2KO mice would increase normal insulin signaling, leading to improved retinal function.

**Methods:**

C57BL6 or β2KO mice were left untreated or treated with etanercept (0.3 mg/kg subcutaneously, 3× a week) for 2 months. Electroretinogram analyses were done before treatment was initiated and after two months of treatment with etanercept on all mice. Western blot or ELISA analyses were done on whole retinal lysates from all four groups of mice for TNFα, suppressor of cytokine signaling 3 (SOCS3), insulin receptor, and apoptotic proteins.

**Results:**

Etanercept significantly reduced TNFα levels in β2KO mice, leading to increased insulin receptor phosphorylation on tyrosine 1150/1151. SOCS3 levels were increased in β2KO mice, which were reduced after etanercept treatment. Pro-apoptotic proteins were reduced in etanercept-treated β2KO mice. Etanercept improved ERG amplitudes in β2KO mice.

**Conclusions:**

Inhibition of TNFα by etanercept protects the retina likely through reduced TNFα-mediated insulin resistance, leading to reduced apoptosis.

## Background

The role of sympathetic nerves in diabetes has not been questioned when focusing on peripheral nerve disease [1]. Work has demonstrated that diabetes and aging can both produce significant remodeling of sympathetic ganglia and neurotransmission [2]. However, the role of sympathetic nerve activity in the retina is less clear. Work by our group demonstrated that loss of dopamine beta hydroxylase (a key enzyme required for the production of norepinephrine) produces substantial changes to the retina [3]. Subsequently, we have shown that β-adrenergic receptors are key to retinal damage, which is similar to retinal changes observed in animal models of diabetic retinopathy [4,5]. Recently, we showed that treatment of β2KO mice with a novel β1/β2-adrenergic receptor agonist, Compound 49b, could prevent impaired insulin receptor signal transduction [6] observed in both diabetic rodent models [7], as well as retinal endothelial [8] and Müller cells [9] grown under hyperglycemia conditions. Retinal endothelial cells express only β1- and β3-adrenergic receptors [10], while retinal Müller cells express both β1- and β2-adrenergic receptors [11]. Therefore, use of the β2-adrenergic receptor knockout mice (β2KO) mice could suggest that the retinal changes observed in these mice are produced by retinal endothelial cells through activation of β1- and/or β3-adrenergic receptor signaling. Since we have shown that retinal endothelial cells have increased apoptosis in response to high glucose and impaired insulin signaling, it is likely that impaired insulin signaling may be key to apoptosis in the retina [8].

In our previous work in retinal endothelial cells, we have reported that high glucose increases TNFα levels, leading to apoptosis of these cells [8]. Others have also reported that increased cytokine levels can lead to apoptosis of retinal endothelial cells [12]. Increased TNFα can produce insulin resistance in multiple ways. The most direct pathway of TNFα-induced inhibition of insulin signaling is through TNFα-mediated phosphorylation of insulin receptor substrate 1 (IRS-1) on serine 307 [13]. Phosphorylation of serine 307 on IRS-1 inhibits the ability of IRS-1 to transmit the insulin phosphorylation signal to Akt, thus blocking normal insulin signal transduction. In addition to actions on IRS-1, TNFα also leads to increased suppressor of cytokine signaling 3 (SOCS3) levels [14,15]. Increased SOCS3 levels can phosphorylate the insulin receptor on tyrosine 960 which blocks the insulin receptor/IRS-1 interaction [16]. We have previously reported that TNFα and SOCS3 are both increased in response to high glucose in retinal endothelial cells, leading to increased phosphorylation of IRS-1^Ser307^ and IR^Tyr960^ [8]. The remaining question was whether this occurred *in vivo* and whether inhibition of TNFα could block all downstream responses to restore normal insulin signal transduction.

The suggestion of inhibition of TNFα as a therapeutic for diabetic retinopathy is not novel. Because TNFα is reported to cause insulin resistance in many other tissues, including adipocytes [13] and myeloid progenitor cells [17], it would be expected that inhibition of TNFα would protect cells and normalize insulin signaling. This was directly tested in chronic studies in TNFα receptor 1- or receptor 2-deficient mice fed 30% galactose for up to 20 months. The authors found that inhibition of TNFα with etanercept (Enbrel), a TNFα receptor antagonist, in galactosemic rats led to reduced pericyte loss and degenerate capillary formation [18]. Additionally, in acute studies of retinopathy-like changes, the authors demonstrated that etanercept suppressed caspase activity and apoptosis in Long Evans rats treated with streptozotocin to make them diabetic [18]. Previously, this group had reported that etanercept reduced leukocyte adhesion in diabetic rats [19]. Therefore, it is clear that inhibition of TNFα is protective against diabetic retinopathy changes; however, the mechanism by which this may occur is unknown.

Our hypothesis was that inhibition of TNFα actions in β2KO mice would restore normal insulin signal transduction, explaining the improvement in markers of diabetic retinopathy reported by others. To test our hypothesis, we treated wildtype mice or β2KO mice with etanercept for two months and measured proteins involved in insulin resistance and signaling, including TNFα, SOCS3, insulin receptor (IR), and apoptotic markers. We found that inhibition of TNFα by etanercept treatment to β2KO mice was able to significantly reduce apoptotic markers through decreased activation of TNFα-mediated insulin resistance.

## Methods

### Mice

All mice experiments, including those for dark-adaptation and tail electrodes for electroretinogram (ERG) analyses, were approved by the Institutional Animal Care and Use Committee at the University of Tennessee Health Science Center (Protocol #1992). β1/β2KO mice (*Adrb1*^*tm1*^*Bkk Adrb2*^*tm1*^*Bkk*/J) were purchased from Jackson Laboratories (Bar Harbor, ME, USA). From the β1/β2KO mice, we bred mice to generate only homozygous β2KO mice. We appreciate that C57BL6 may not be the ideal wildtype control, but the original β1/β2KO mice were from a mixed background containing C57BL6, therefore we chose the C57BL6 for wildtype. Since we use these mice at three months of age, other issues from the C57BL6 background should be minimized. We have previously published use of this animal model and genotyping [5] to demonstrate that neuronal markers of diabetic retinopathy are present, as well as increased apoptosis in the retina.

### Etanercept (Enbrel) treatment

A subset of the β2KO mice were administered Enbrel by subcutaneous injection 0.3 mg/kg, 3×/week) [19]. After the final treatment, C57BL6 control, C57BL6+ etanercept, β2KO mice, and β2KO mice + etanercept (five mice of each gender in each group) were dark-adapted for ERG analyses prior to sacrifice by ketamine and xylazine overdose.

### Electroretinogram

Prior to sacrifice for morphological and biochemical analyses, animals were subjected to ERG analyses to evaluate the changes in the electrical activity of the retina as we have done previously [5,7]. After dark-adaptation overnight, ERG responses were recorded from both eyes together using platinum wire corneal electrodes, forehead reference electrode, and ground electrode in the tail. Pupils were fully dilated using 1% tropicamide solution (Alcon, Ft. Worth, TX, USA). Methylcellulose (Celluvise; Allergan, Irvine, CA, USA) drops were applied as well to maintain a good electrical connection and body temperature was maintained at 37°C by a water-based heating pad. ERG waveforms were recorded with a bandwidth of 0.3 to 500Hz and sampled at 2 kHz by a digital acquisition system and were analyzed using a custom-built program (MatLab, Mathworks, Natick, MA, USA). Statistics were done on the mean ± SD amplitudes of the A- and B-wave of each treatment group.

### Western blot analysis

Equal amounts of protein from the tissue extracts were separated on the pre-cast tris-glycine gel (Invitrogen, Carlsbad, CA, USA), blotted onto a nitrocellulose membrane. After blocking in TBST (10 mM Tris–HCl buffer, pH 8.0, 150 mM NaCl, 0.1% Tween 20) and 5% (w/v) BSA, the membrane was treated with appropriate primary antibodies followed by incubation with secondary antibodies labeled with horseradish peroxidase. Antigen-antibody complexes were detected by a chemiluminescence reagent kit (Thermo Scientific, Waltham, MA, USA). Primary antibodies used were phosphorylated Akt (Serine 473), Akt, Bax, Bcl-xL, Cytochrome C, SOCS3, phosphorylated insulin receptor (tyrosine 1150/1151), insulin receptor (all purchased from Cell Signaling, Danvers, MA, USA), insulin receptor phosphorylated on Tyr960 (Cell Applications, San Diego, CA, USA), and beta actin (Santa Cruz Biotechnology Inc., Santa Cruz, CA, USA).

### ELISA analysis

A cleaved caspase 3 ELISA (Cell Signaling, Danvers, MA, USA) was used to measure levels of the active apoptotic marker in whole retinal lysates. TNFα protein concentrations were measured using a TNFα ELISA (ThermoFisher, Pittsburgh, PA, USA). For cleaved caspase 3 ELISA analyses, equal protein was loaded (50 μg) into all wells to allow for comparisons based on optical density (OD). For the TNFα ELISA, 50 μl protein was loaded into all wells, with analyses for concentrations based on a standard curve.

### Statistics

Statistical analyses were done using Prism software (GraphPad, La Jolla, CA, USA). Analyses were done using a Kruskal-Wallis test, followed by Dunn’s test. Data are presented as mean ± SEM. For Western blots, a representative blot is presented.

## Results

### Etanercept treatment restores normal insulin receptor phosphorylation, while reducing IRS-1^Ser307^ levels

**Figure 1:**
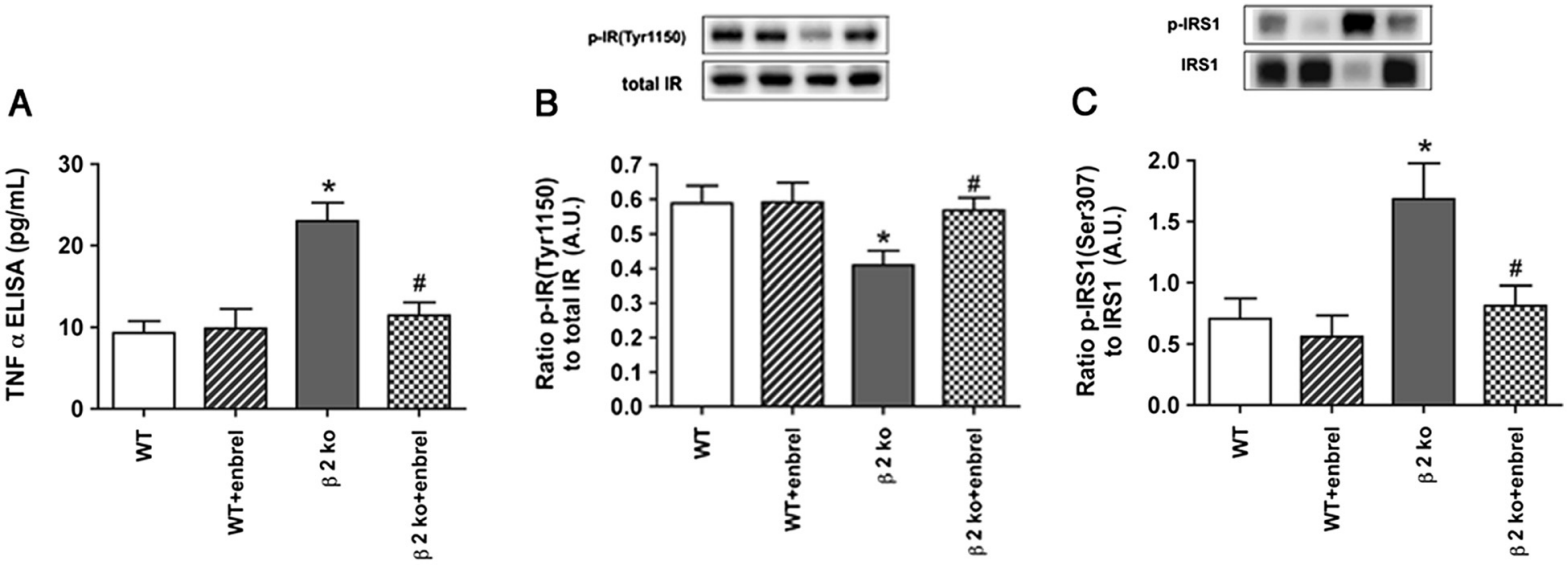
**Enbrel reduced TNFα and IRS-1** ^**Ser307**^ **phosphorylation, while increasing insulin receptor autophosphorylation.** In all panels, data shows results for wildtype (WT) and β2KO (β2KO) mice only or WT and β2KO mice treated with etanercept. **(A)** Demonstrates that etanercept was effective in reducing TNFα levels, which are increased in β2KO only mice. **(B)** Shows that insulin receptor autophosphorylation sites tyrosine 1150/1151 are reduced in β2KO mice, which are restored after etanercept treatment. **(C)** Demonstrates that IRS-1^Ser307^ is reduced after etanercept treatment to β2KO mice. *P < 0.05 versus WT. #*P* < 0.05 versus β2KO only. Data are mean ± SEM. N = 5 mice for all groups.

To insure that treatment with etanercept was able to significantly reduce TNFα levels in β2KO mice, we performed an ELISA analysis to show that β2KO mice have increased levels of TNFα, which were significantly reduced by etanercept treatment (Figure 1A). Since TNFα is a key player in insulin resistance, we wanted to investigate whether blockade of TNFα actions in β2KO mice could prevent these deleterious effects. Etanercept treatment to β2KO mice was able to significantly increase insulin receptor autophosphorylation on tyrosine 1150/1151 (Figure 1B). Since TNFα preferentially phosphorylates IRS-1 on serine 307 to inhibit insulin signal transduction [13], we measured IRS-1^Ser307^ phosphorylation after etanercept treatment. Data showed that etanercept significantly reduced IRS-1^Ser307^ phosphorylation in β2KO mice (Figure 1C), suggesting that treatment with etanercept prevented TNFα-induced IRS-1 phosphorylation.

### Etanercept reduced SOCS3 levels, leading to decreased IR^Tyr960^ phosphorylation

**Figure 2:**
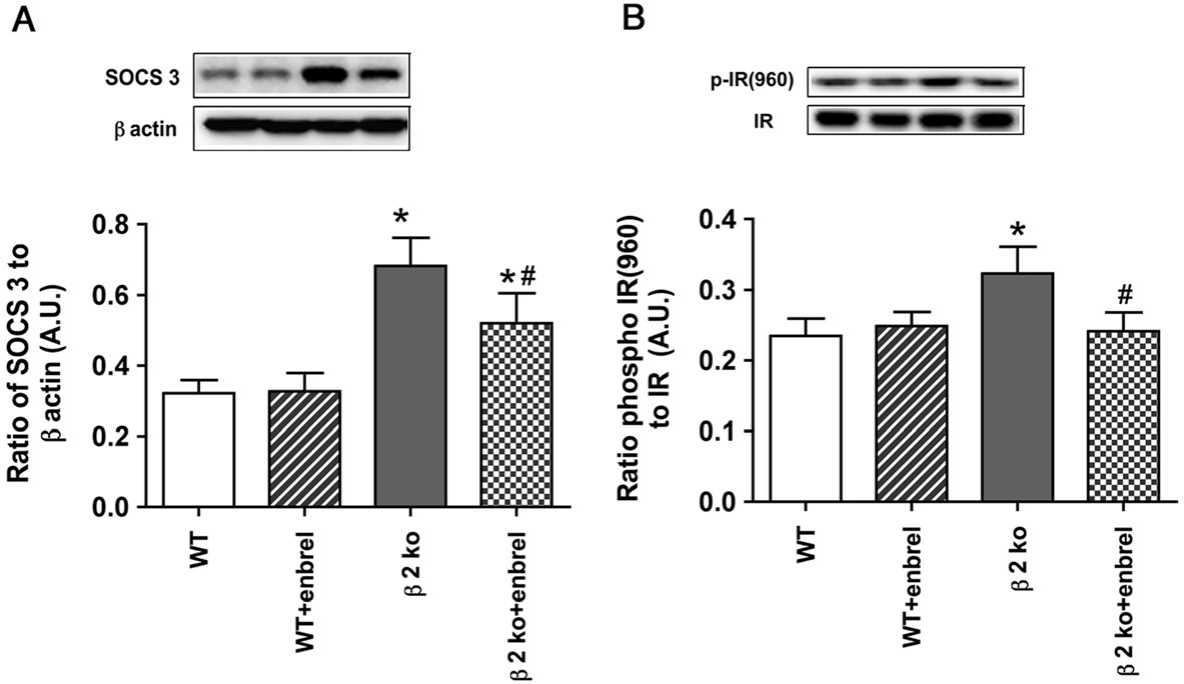
**Inhibition of TNFα with etanercept reduces suppressor of cytokine signaling 3 (SOCS3) and IR** ^**Tyr960**^ **levels.** In all panels, data shows results for wildtype (WT) and β2KO (β2KO) mice only or WT and β2KO mice treated with etanercept. **(A)** Western blot data on SOCS3 levels demonstrating that etanercept reduced SOCS3 levels in β2KO mice. **(B)** Western blot data on insulin receptor phosphorylation on tyrosine 960, showing that IR^Tyr960^ is reduced after etanercept treatment. *P < 0.05 versus WT. #*P* < 0.05 versus β2KO only. Data are mean ± SEM. N = 5 mice for all groups.

We have previously reported that β2KO mice have increased SOCS3 levels and IR^Tyr960^ phosphorylation [5], which are both associated with increased TNFα levels and insulin resistance [15,16]. Figure 2 demonstrated that β2KO mice have increased SOCS3 (A) and IR^Tyr960^ phosphorylation (B), as we reported previously. Additionally, Figure 2 showed that inhibition of TNFα with etanercept was able to significantly reduce both SOCS3 and IR^Tyr960^ activity, suggesting that etanercept may prevent insulin resistance in the retina.

#### Pro-apoptotic factors are reduced after etanercept treatment to β2KO mice

**Figure 3:**
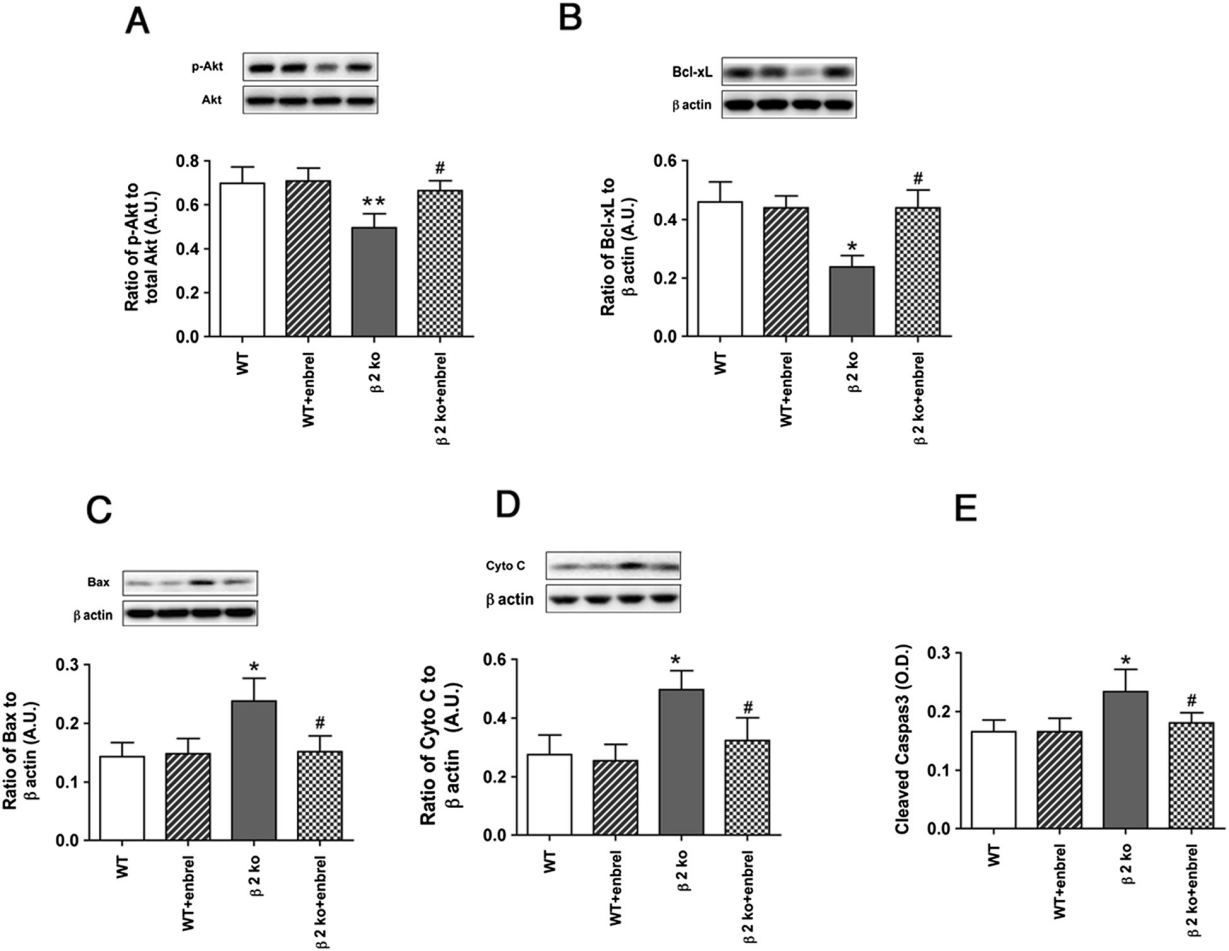
**Etanercept treatment reduces pro-apoptotic markers.** In all panels, data shows results for wildtype (WT) and β2KO (β2KO) mice only or WT and β2KO mice treated with etanercept. **(A)** and **(B)** show that etanercept increases anti-apoptotic markers Akt **(A)** and Bcl-xL **(B)**, while **(C-E)** demonstrate the etanercept decreased pro-apoptotic markers Bax **(C)**, Cytochrome C **(D)** and cleaved caspase 3 **(E)**. *P < 0.05 versus WT. #*P* < 0.05 versus β2KO only. Data are mean ± SEM. N = 5 mice for all groups.

Since prevention of apoptosis is a key goal of the insulin signaling pathway, we measured key pro-apoptotic (Figure 3C-E) and anti-apoptotic markers (Figure 3A-B) in wildtype and β2KO mice. While etanercept had no effects on wildtype mice, etanercept treatment significantly increased levels of key anti-apoptotic markers, Akt (A) and Bcl-xL (B), while decreased pro-apoptotic markers, Bax (C), Cytochrome C (D), and cleaved caspase 3 (E). Taken together, the data suggest that β2KO mice have impaired insulin signaling, which is restored after inhibition of TNFα through etanercept therapy.

### Etanercept treatment improves ERG amplitudes

**Figure 4:**
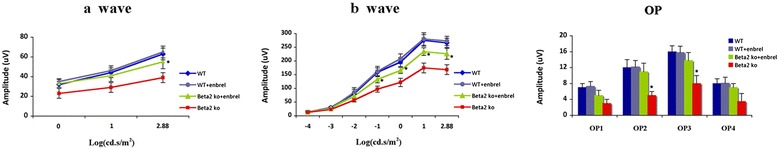
**Etanercept improved amplitudes of all electroretinogram (ERG) waves in β2KO mice.** In all panels, data shows results for wildtype (WT) and β2KO (β2KO) mice only or WT and β2KO mice treated with etanercept. In mice treated with etanercept for two months, the ERG amplitudes for A-wave (left), B-wave (middle) and oscillatory potentials (right) were significantly increased over β2KO only mice. Data are mean ± SD for ERG. N = 5 mice for all groups.

We have previously reported that β2KO mice have reduced amplitudes of the A-wave, B-wave and oscillatory potentials [5], which was improved with treatment with Compound 49b (Jiang *et al*., in press). In this study, we found a similar reduction in ERG amplitudes in β2KO mice compared to wildtype mice (Figure 4, red line), which was resolved with treatment with etanercept for two months (Figure 4, green line). Combining the cell signaling data with functional data, this work demonstrated that TNFα inhibition is effective in preventing insulin resistance in the retina, leading to normal retinal function.

## Discussion

The goal of this study was to demonstrate that TNFα plays a role in insulin resistance in β2KO mice. Since others had reported that etanercept was effective in eliminating diabetic-like changes in the rodent eye [18,19], we wanted to determine if inhibition of TNFα with etanercept in β2KO mice could restore normal insulin signal transduction. We have previously demonstrated that TNFα is key to inhibition of insulin signaling in both retinal endothelial cells (REC) [8] and Müller cells [20] grown in a high glucose concentration, leading to increased apoptosis. In this data set, we demonstrate that etanercept is effective at reducing TNFα-mediated insulin resistance. Two months of etanercept treatment, used at the same treatment regimen as that used in humans with rheumatoid arthritis [19], led to a significant decrease in TNFα in β2KO mice when compared to untreated β2KO mice. Since we have reported that one pathway by which TNFα can mediate insulin resistance in REC is through phosphorylation of IRS-1^Ser307^ [8], we measured this phosphorylation site *in vivo* and found that etanercept reduced IRS-1^Ser307^ in the β2KO mice. Another potential pathway by which TNFα can mediate impaired insulin signaling is through activation of SOCS3 [15]. Etanercept was able to significantly reduce SOCS3 in β2KO mice, leading to decreased IR^Tyr960^. Blockade of IR^Tyr960^ phosphorylation can promote normal insulin signaling as IR^Tyr960^ inhibits the interaction between insulin receptor and IRS-1 [16].

In addition to blockade of TNFα-mediated impairment of insulin signal transduction, etanercept also promoted phosphorylation of insulin receptors on tyrosine 1150/1151 in the β2KO mice, which are autophosphorylation sites, leading to promotion of insulin signaling. We have previously reported that insulin receptor phosphorylation is reduced in β2KO mice, which appeared to be localized to the inner retina and ganglion cell layer [5]. Maintenance of normal insulin signal phosphorylation by etanercept likely led to increased Akt activity and decreased cleaved caspase 3 observed in the β2KO mice. The reduced apoptosis and improvement in insulin signaling in the inner retina may be correlated to the improvement in the ERG after etanercept treatment.

We focus on our previous work in REC, as β2KO mice treated with etanercept likely represent effects of TNFα inhibition on REC only, as we have previously reported that β2-adrenergic receptors are key for TNFα actions in Müller cells [20,21]. Thus, use of the β2KO mice allows us to dissect TNFα-mediated effects on insulin signaling in REC versus Müller cells’ actions *in vivo*. Data from etanercept-treated β2KO mice suggest that maintenance of normal insulin signaling through TNFα inhibition can reduce inner retinal apoptosis and improve retinal function.

Others have reported that TNFα inhibition using etanercept is effective at reducing diabetic-like changes in the retina [18,19]. This study provided novel information on potential reasons for the improvement in diabetic-like changes; that is, reduced TNFα-mediated impairment of insulin signaling. Data demonstrate that inhibition of TNFα in β2KO mice led to improved insulin receptor phosphorylation on tyrosine 1150/1151, as well as increased anti-apoptotic proteins. Use of etanercept in β2KO mice allowed us to dissect TNFα-mediated impairment of insulin signal transduction in REC versus Müller cells. Taken together, the present study provides cellular signaling pathways associated with the beneficial effects of etanercept on β2KO mice.

## Abbreviations

β2KO: β2-adrenergic receptor knockout mice
ELISA: enzyme-linked immunosorbent assay
ERG: electroretinogram
IR: insulin receptor
IRS-1: insulin receptor substrate 1
REC: retinal endothelial cells
SOCS3: suppressor of cytokine signaling 3
TNFα: tumor necrosis factor alpha
WT: wildtype
OD: optical density
